# Efficacy and Safety Assessment of T. Angelica Herbal Tonic, a Phytomedicinal Product Popularly Used in Nigeria

**DOI:** 10.1093/ecam/nep161

**Published:** 2011-03-09

**Authors:** Charles O. Esimone, Peter A. Akah, Chukwuemeka S. Nworu

**Affiliations:** ^1^Department of Pharmaceutics, Faculty of Pharmaceutical Sciences, University of Nigeria, Nsukka 410001, Enugu State, Nigeria; ^2^Department of Pharmacology & Toxicology, Faculty of Pharmaceutical Sciences, University of Nigeria, Nsukka 410001, Enugu State, Nigeria

## Abstract

T. Angelica Herbal Tonic (TAHT) is a herbal product indicated for indigestion and constipation and highly patronized in Nigeria. In this study, the efficacy and safety of the herbal tonic in relation to the label claims were assessed. The effect on peristalsis in mice was evaluated by the charcoal meal model and *in vitro* using guinea pig ileum. The effects of TAHT on behavior, fertility, birth and organ weights were also determined. Teratogenic potential and reproductive toxicity were studied in pregnant rats. Acute toxicity studies showed that at doses above 5000 mg kg^−1^, the herbal tonic did not cause lethality and produced no signs of intoxication in mice. The study did not show any gross behavioral changes in mice treated with 1000 mg kg^−1^ of TAHT as compared with the negative control treatment. TAHT (400 mg kg^−1^) exhibited a dose-dependent enhancement in the gastrointestinal tract motility in mice when compared with the negative control. At concentrations up to 300 **μ**g mL^−1^, TAHT did not cause any significant effect on acetylcholine, histamine and nicotine-evoked contractions of guinea pig ileum preparation. It took an average of 31.25 ± 4.52 days for the TAHT-treated animals to litter, which is significantly (*P* < .05) different from the 55 ± 4.51 days recorded for the control treatment group. TAHT exhibited a modest fertility-promoting effect and showed lack of abortifacient and teratogenic properties in the study. Generally, the results of this study showed some favorable pharmacological effects of TAHT in animals which may authenticate some of the label claims.

## 1. Introduction

Historically, herbal medicine has been an important component of healthcare all over the world. With the advances in medical and biological sciences that resulted in the introduction of promising synthetic orthodox therapies for many conditions, the use of herbal medicine declined in the 20th century. Lately, however, there has been a resurgence of interest in the use of phytomedicinal products in the treatment of diseases [1, 2]. The World Health Organization (WHO) has documented the rapidly growing interest and economic importance of Traditional Medicine (TM) in health systems all over the world.

In Africa, a WHO report estimated that up to 80% of the population use TM to help meet their health care needs either alone or in combination with orthodox therapies [3, 4]. Asian and Latin American populations continue to use TM as a result of historical circumstances and cultural beliefs. In China, TM accounts for *∼*40% of all health care services delivered. In many developed countries, complementary and alternative medicines (CAMs) is also becoming more and more popular. The percentage of the population which has used CAM at least once is 48% in Australia, 70% in Canada, 42% in USA, 38% in Belgium and 75% in France. In many parts of the world, expenditure on TM/CAM is not only significant, but also growing rapidly. In Australia, Canada and the UK, annual CAM expenditure is estimated at US$ 80, US$ 2400 and US$ 2300 million, respectively [5, 6].

With the increasing popularity and patronage of commercially promoted herbal medications, the need for the assessment of the safety and quality of these products is increasingly being felt. The safety evaluation of herbal products is even more important since the popular belief that herbal therapies are without untoward effects have often been proven incorrect [7, 8]. Assurances of safety, efficacy and quality of herbal medicines have been limited by lack of research methodology, inadequate evidence base for TM/CAM therapies and products, lack of international and national standards, lack of adequate regulation and registration of herbal medicines, lack of registration of TM/CAM providers and inadequate support for such research efforts [9, 10].

T. Angelica Herbal Tonic (TAHT) is an amber-colored liquid preparation with slight bitter taste, packaged in 750 mL amber colored plastic bottle. TAHT is promoted as a mild laxative with: “aids digestion”, “relieves constipation”, “eliminates bloatness”, and “enhances bowel cleansing” as key label indications. TAHT is labeled to contain *Doundake* root (3.71%) and *Cassia acutifolia* (1.0%) as the active ingredients and Kalii nitras (0.073%), Methyl paraben (0.2%), Propyl paraben (0.02%) and deionized water as inactive ingredients. This herbal product is highly patronized in Nigeria and is claimed to be useful for a variety of purposes by the public. This stimulated the interest in conducting an investigation into the safety and efficacy of the products in relation to the claim of this product using laboratory animal models.

## 2. Materials and Methods

### 2.1. The Product

TAHT is an amber colored liquid preparation with a slightly bitter taste. It is packaged in an amber plastic bottle of 750 mL capacity, closed with a tamper-proof aluminum screw cap. Each 150 mL of the preparation contains Doundake root obtained from *Nauclea latifolia* (5.56 g, equivalent to 2.36 mg/mL total alkaloid) and *C. acutifolia* leaf (1.5 g, equivalent to 3.075% sennoside B). In this study, the dosing of the TAHT to animals was based on the total active ingredient content per milliliter of the tonic.

Permission to undertake the study was obtained from the manufacturer, HErBALINE Nigeria Ltd, and samples used in the study were obtained from the manufacturer's direct supply chain.

### 2.2. Animals

Adult albino rats (100–200 g), albino mice (21–25 g) and guinea pigs (220–280 g) of both sexes, obtained from the Laboratory Animal Facility of the Department of Pharmacology and Toxicology, University of Nigeria, Nsukka were used in the study. The animals were housed under standard conditions (25 ± 2°C and 12-h light/dark cycle). The rats and mice were maintained on standard pellets (Livestock feed), while guinea pigs were fed with guinea grass (*Panicum maximum L*.). All animals were allowed unrestricted access to drinking water. The use and care of laboratory animals in the study were in accordance with the ethical guidelines contained in the European Convention for the Protection of Vertebrate Animals used for Experimental and Other Scientific Purposes (EEC Directive of 1986; 86/609/EEC) as amended in the European Treaty Series (ETS no. 170) of 2005.

### 2.3. Acute Toxicity Determination

The acute toxicity study of TAHT was assessed by oral administration in albino mice using the Lorke (1983) method [11]. Briefly, the tests involved two phases. The first phase is the determination of the toxic range. The mice were placed in three groups (*n* = 3) and were administered TAHT (10, 100 and 1000 mg kg^−1^) orally. The treated mice were observed for 24 h for the number of deaths. The death pattern in the first phase determined the doses used for the second phase according to the Lorke (1983) estimation [11]. In this phase, four groups (*n* = 3) of mice were used for each dose. Each group received different doses of the TAHT (p. o.) as predetermined in the first phase and the animals were observed for lethality or signs of acute intoxication for 24 h. The LD_50_ was calculated as the geometric mean of the highest nonlethal dose and the least toxic dose.

### 2.4. Gastrointestinal Tract Motility

The primary indication of the herbal tonic is for bowel cleansing, relief of constipation and mild purgation. This necessitated the investigation of the effect of the TAHT on gastrointestinal (GI) motility. Sixteen albino mice of either sex (20–30 g), randomly divided into four groups (*n* = 4) were used in the study. The animals were starved for 24 h prior to the experiment, but were allowed unrestricted access to drinking water. The first two groups received 200 and 400 mg kg^−1^, respectively, while the 3rd and 4th groups received carbachol (40 mg kg^−1^) and normal saline (10 mL kg^−1^). After 5 min of drug administration, 0.5 mL of 5% charcoal dispersion in tragacanth mucilage was administered orally to each animal. The animals were sacrificed 30 min later and the abdomen opened. The percentage distance traveled by the charcoal plug in the small intestine (from the pylorus to the caecum) in the treatment groups were determined and compared with the normal saline-treated group used as the negative control.

### 2.5. Neuropharmacological Activity

Ten mice placed into two groups (*n* = 5) were used in the study. The first group was given TAHT (1000 mg kg^−1^, p.o.) and the second group served as the control and was given normal saline (20 mL kg^−1^, p.o.). The treated animals were observed for 24 h and behavioral parameters (including awareness, mood, motor activity, central activity, muscle tone, reflexes and autonomic effects) were monitored.

### 2.6. Reproductive and Developmental Toxicity Investigation

These studies assessed TAHT for abortifacient, teratogenic and deleterious developmental effects. Virgin rats were divided into two groups (*n* = 5) and acclimatized for 15 days. During this time, the test group was given oral dose of TAHT (500 mg kg^−1^) daily (*∼*10 times the daily recommended dose of 60 mg kg^−1^), while the control group daily received 5 mL kg^−1^ of distilled water orally. The animals were monitored for normal growth and development. After this period, two mature male rats of proven fertility (based on earlier ability to cause pregnancies) were introduced into each cage and the number of days that elapsed before delivery was recorded for the rats in each group. During gestation, the rats were monitored for incidences of possible spontaneous abortion. The birth weight of litters, sex (determined 3 weeks after birth) and total number of litters were recorded. The litters were also observed for incidences of malformation. Daily oral administration of TAHT continued while animals were lactating. The litters were also observed for growth and development for a period of 3 weeks. At the end of the study, the adult female rats were sacrificed and gross morphological examinations carried out on the major organs (heart, lungs, spleen, liver and kidney); the weights of the organs were also determined.

### 2.7. In Vitro Pharmacological Experiments

#### 2.7.1. Guinea Pig Ileum Activity

This study was conducted to determine the effects of TAHT on the smooth muscle of GIT. Segments of ileum isolated from freshly sacrificed guinea pig were suspended in 30 mL organ bath containing Tyrode's solution at 37 ± 1°C aerated with air. The effect of TAHT on the isolated tissue was studied according to classical methods. Increasing concentrations of TAHT up to 300 *μ*g mL^−1^ were added into the organ bath to determine its effect on isolated ileum. The effect of TAHT on contractions produced by standard agonists: acetylcholine (0.3 *μ*g mL^−1^), histamine (0.06 *μ*g mL^−1^) and nicotine (30 *μ*g mL^−1^) were also studied.

#### 2.7.2. Gravid Rat Uterus Activity

The possible effect of TAHT on intrinsic rhythmic contraction of isolated gravid uterus, and therefore the assessment of possible tocolytic or abortifacient properties was also determined. The two horns of uterus isolated from a freshly sacrificed gravid rat were cut longitudinally into strips. A strip was mounted in De Jalon's solution and the effect of increasing concentrations of TAHT on the spontaneous rhythmic contraction of the uterine strip was monitored.

### 2.8. Statistical Analysis

Data were analyzed by analysis of variation, ANOVA using SPSS (version 11) and differences between paired observations were regarded as significant at *P* ≤ .05.

## 3. Results

### 3.1. Toxicity of TAHT

At doses above 5000 mg kg^−1^, the herbal tonic did not cause lethality and produced no signs of acute intoxication in the mice. Therefore, TAHT could generally be regarded as safe. The study did not show any gross behavioral changes in mice treated with 1000 mg kg^−1^ of TAHT as compared with the negative control treatment (Table 1).

### 3.2. TAHT Improves GIT Motility

The TAHT exhibited a dose-dependent enhancement in the GI tract (GIT) motility in mice. The increase in the intestinal motility induced by the higher dose (400 mg kg^−1^) was significant (*P* < .05) when compared with the negative control; however, the effect was slightly below the increase in peristalsis produced by carbachol 40 mg kg^−1^, a standard muscarinic agonist (Table 2). Addition of TAHT at concentrations up to 300 *μ*g mL^−1^ did not produce any significant effect on acetylcholine, histamine and nicotine-evoked contractions of guinea pig ileum preparation.

### 3.3. Reproductive and Developmental Activity of TAHT

**Figure 1:**
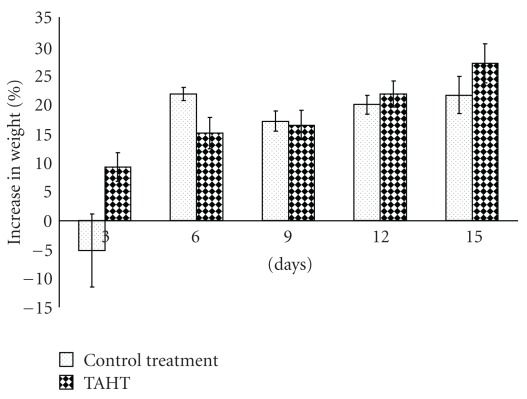
Increase in weight of virgin rats during the administration of THAT before mating.

**Figure 2:**
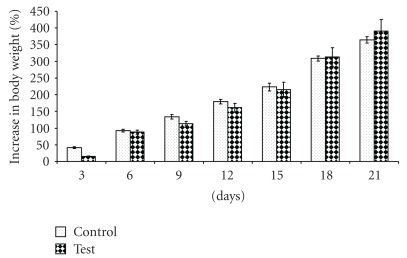
Percentage increase in the weight of the litters over a 21-day monitoring period.

There was no significant difference (*P* > .05) in the increase in the weight of the virgin rats treated with TAHT and the control treatment after a 15-day monitoring period (Figure 1). It also took an average of 31.25 ± 4.52 days for the test (treated) animals to litter. This is significantly (*P* < .05) different from the 55 ± 4.51 days that elapsed before delivery was recorded for the control treatment group. By the 43rd day of mating, 80% of the rats in the test group had delivered while none delivered in the control group (Table 3). The mean weight of the litters in the control group (5.95 ± 0.09 g) was found to be significantly higher (*P* < .05) than that of the test group (4.81 ± 0.19 g) (Figure 2). The mean number of litters was 7.50 ± 0.05 and 5.25 ± 0.95 in the test and control groups, respectively (Table 3). Subsequent monitoring of the litters in both groups showed that there was no significant difference in the percentage increase in weight over the 21-day observation period (Table 3 and Figure 2), indicating lack of developmental effect on lactating animals (Table 3).

**Figure 3:**
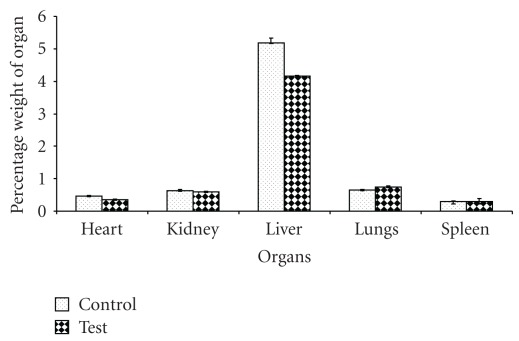
Percentage weight of the organs (relative to the body weight) of the rats after sacrifice.

No spontaneous abortion or malformation was noted in any of the pregnant rats and litters in both groups. The results indicate that TAHT lacks abortifacient and teratogenic properties. Gross morphological examination of the major organs showed no difference in the organs from both the control and the test animals. However, significant differences (*P* < .05) were noted in the mean weight of the heart and liver, where higher mean values were recorded for the control group animals (Figure 3).

### 3.4. Activity of TAHT on Uterine Strips

**Figure 4:**
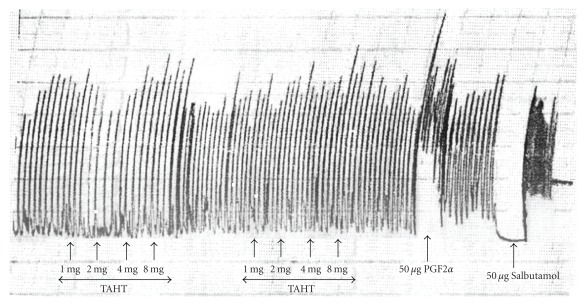
The effect of THAT (cumulative) on intrinsic contractility of the gravid rat uterus. A sample tracing showing the treatment of rat uterine strips with increasing amount of (1–8 mg) of THAT, PGF2*α* and Salbutamol. THAT did not affect the frequency and amplitude of uterine strips inherent contractility.

TAHT did not modify the intrinsic contractility of isolated uterine preparations. In the experiment, salbutamol (a tocolytic agent) completely relaxed the uterine strip while prostaglandin F_2*α*_ (PGF_2*α*)_, a typical abortifacient, also caused sustained increase in amplitude and frequency of the uterine muscle strip contraction (Figure 4).

## 4. Discussion

In the study on mice, TAHT with LD_50_  >  5000 mg kg^−1^ could be considered safe [11]. Although safety studies in animal models could often be extrapolated to humans, it does not necessarily follow that such products would be completely safe in humans. Therefore, actual controlled clinical trials on human subjects would be more definitive evidence on safety. TAHT did not cause the contraction of guinea pig ileum preparation and did not produce any significant effect on acetylcholine, histamine and nicotine-evoked contractions of the tissue. This result suggests that the pro-kinetic effect of TAHT on GIT may not be mediated by a direct effect on muscarinic, histaminergic or nicotinic receptors stimulation. Although the laxative effect of TAHT recorded in the whole animal experiments could support the benefits claimed in the use of TAHT for indigestion, constipation and bloating of stomach, the exact mechanism by which TAHT induces increase in the intestinal motility is not clear from the study.

The lower mean birth weights recorded in the group treated with TAHT could be ascribed to the higher number of litter size (a total of 30 for test group and 21 for control group), since higher multiple pregnancy could lead to lower mean birth weights. Subsequent monitoring of the litters in both groups showed that there was no significant difference in the percentage increase in weight over 21-day observation period, indicating lack of developmental effect on lactating animals. Although the study demonstrates some fertility promotion effect of TAHT, more studies will be required to definitively confirm any possible effect on sexuality, fertility and fertility hormones. The study also showed that TAHT lacks abortifacient and teratogenic effects.

TM and CAM are now attracting increasing attention and many governments are now according relevance to CAM in the provision of health care all over the world [12]. Some important issues must be addressed if the potentials of TM/CAM are to be developed successfully. It is known that herbs in their natural state vary in potency and may contain multiple pharmacological substances which can cause undesirable effects. In most instances, herbal medicines are not made with isolated and purified active substance that will permit standardization of dosages, and testing for safety and efficacy before approval [13]. Some toxic reactions to the use of herbal remedies are related to improper preparation, excessive quantities of some herbs, substitution of a herb with related herbs believed to possess similar effects and the effect of using herbal medications concomitantly with other pharmacologic products [14, 15].

In this study, a scientific assessment of a highly patronized herbal preparation marketed and used in Nigeria was undertaken. The results of this study show some level of efficacy and safety, which are related to some of the claimed indications of the phytomedicine. The results also showed some favorable pharmacological effects in animal models, which authenticates some of the label claims. However, further exhaustive studies, especially in human volunteers, would be necessary to succinctly validate the safety and efficacy of this herbal tonic.

## Funding

Personal finances of the authors.

## Figures and Tables

**Table 1:** The effect of acute dose of TAHT (1000 mg kg^−1^) on the behavioral pattern of mice.

Bahavioral activity	Description	Control treatment score^a^	Test treatment scores^a^
Awareness	Alertness	4	4
	Passivity	0	1
Mood	Grooming	4	4
	Vocalization	0	0
	Fearfulness	0	0
	Aggressiveness	0	0
Motor activity	Inquisitive behavior in unfamiliar environment	0	0
	Touch response	4	4
	Pain response	4	4
Central activity	Response to noise	4	4
	Tremor	0	0
	Convulsion	0	0
Muscle tone	Limb tone	4	4
	Grip strength	4	4
Reflexes	Pinna reflex	4	4
	Cornea reflex	4	4
Autonomic effects	Respiratory rate	4	4
	Piloerection	0	0
	Writhing	0	0

^a^The scores were recorded on a scale of 0–8 with a base score of a normal response as 4.

**Table 2:** Effects of TAHT on gastrointestinal motility in mice.

Agents	Dose (mg kg^−1^)	Percentage of intestinal transit
Carbachol	40	73.03 ± 5.72*
Normal saline	20 mL kg^−1^	56.02 ± 6.88
TAHT	200 mg kg^−1^	65.90 ± 4.02
TAHT	400 mg kg^−1^	70.93 ± 2.71*

Albino mice were placed randomly into four groups and were starved for 24 h prior to the experiment. The first two groups received 200 and 400 mg kg^−1^ of the TAHT, respectively, while the 3rd and 4th groups received carbachol (40 mg kg^−1^) and normal saline (10 mL kg^−1^). After 5 min of drug administration, 0.5 mL of 5% charcoal dispersion in tragacanth mucilage was administered orally to each animal. The animals were sacrificed 30 min later and the percentage of distance traveled by the charcoal plug in the small intestine (from the pylorus to the caecum) were determined and compared with the negative control.

*Significant *P* < .05 versus control.

**Table 3:** Summary of reproductive effects of TAHT in experimental rats.

Parameter	Control group	Test group
Number of virgin rats	5	5
Percentage of pregnancy at the end of experiment	80	80
Percentage of pregnant by the 43rd day	0	80
Percentage of pregnant by the 65th day	80	80
Mean number of days elapsed before delivery	55.00 ± 4.71	31.25 ± 4.52*
Total litter size	21	30
Average litter size	5.25 ± 0.95	7.50 ± 0.50
Number of male litters	10	16
Number of female litters	11	14
Average birth weight (g)	5.95 ± 0.10	4.81 ± 0.19*
Percentage increase in weight 21 days after birth	364.53 ± 10.01	390.74 ± 34.47

Percent weight of major organs (relative to body weight)		
(i) Heart	0.463 ± 0.024	0.358 ± 0.017*
(ii) Kidney	0.639 ± 0.037	0.595 ± 0.020
(iii) Lung	0.646 ± 0.020	0.750 ± 0.022
(iv) Spleen	0.297 ± 0.024	0.304 ± 0.008
(v) Liver	5.185 ± 0.147	4.17 ± 0.152*

Duration of pregnancy (days) in each of the four pregnancies achieved in both groups (counting from the start of mating)		
(i) First animal	46	21
(ii) Second animal	48	30
(iii) Third animal	61	31
(iv) Fourth animal	65	43

*Significant *P* < .05 versus control.
